# Calcium sensors of ciliary outer arm dynein: functions and phylogenetic considerations for eukaryotic evolution

**DOI:** 10.1186/s13630-015-0015-z

**Published:** 2015-04-30

**Authors:** Kazuo Inaba

**Affiliations:** Shimoda Marine Research Center, University of Tsukuba, 5-10-1 Shimoda, Shizuoka, 415-0025 Japan

**Keywords:** Sperm, Opisthokont, Bikont, Calaxin, Neuronal calcium sensor, Excavate, Algae, Excavates, Eukaryote, Evolution, Fertilization

## Abstract

The motility of eukaryotic cilia and flagella is modulated in response to several extracellular stimuli. Ca^2+^ is the most critical intracellular factor for these changes in motility, directly acting on the axonemes and altering flagellar asymmetry. Calaxin is an opisthokont-specific neuronal calcium sensor protein first described in the sperm of the ascidian *Ciona intestinalis*. It binds to a heavy chain of two-headed outer arm dynein in a Ca^2+^-dependent manner and regulates ‘asymmetric’ wave propagation at high concentrations of Ca^2+^. A Ca^2+^-binding subunit of outer arm dynein in *Chlamydomonas reinhardtii*, the light chain 4 (LC4), which is a Ca^2+^-sensor phylogenetically different from calaxin, shows Ca^2+^-dependent binding to a heavy chain of three-headed outer arm dynein. However, LC4 appears to participate in ‘symmetric’ wave propagation at high concentrations of Ca^2+^. LC4-type dynein light chain is present in bikonts, except for some subclasses of the Excavata. Thus, flagellar asymmetry-symmetry conversion in response to Ca^2+^ concentration represents a ‘mirror image’ relationship between *Ciona* and *Chlamydomonas*. Phylogenetic analyses indicate the duplication, divergence, and loss of heavy chain and Ca^2+^-sensors of outer arm dynein among excavate species. These features imply a divergence point with respect to Ca^2+^-dependent regulation of outer arm dynein in cilia and flagella during the evolution of eukaryotic supergroups.

## Review

Cilia and flagella are eukaryotic machineries for cell motility propelled by the propagation of bending waves. The internal cytoskeletal structures, called axonemes, are constructed from 9+2 microtubules with axonemal dyneins and regulatory structures such as the central apparatus and radial spokes [1]. These structures are well conserved in all eukaryotes except those that have lost them during evolution. Ciliary and flagellar bend propagations are generated by propagation of sliding of doublet microtubules by axonemal dyneins [2-7]. The propulsive forces generated by bend propagation of cilia and flagella are considered an adaptation for efficient movements by generating fluid flow in microenvironments with low Reynolds numbers [8].

Motility of cilia and flagella is modulated by several extracellular stimuli to enable directed and harmonious movement of cells and tissues. Ca^2+^ is an important factor for these modulations. Here, I first introduce the diversified roles of Ca^2+^ in ciliary and flagellar motility over several eukaryotes and then focus on the Ca^2+^ sensors that directly regulate the motile machinery, the axonemes. In addition, I present a phylogenetic analysis of Ca^2+^ sensors, demonstrating the evolution of Ca^2+^ sensors and proposing a pathway of eukaryotic evolution.

### Cilia and flagella change their motility in response to Ca^2+^

**Figure 1:**
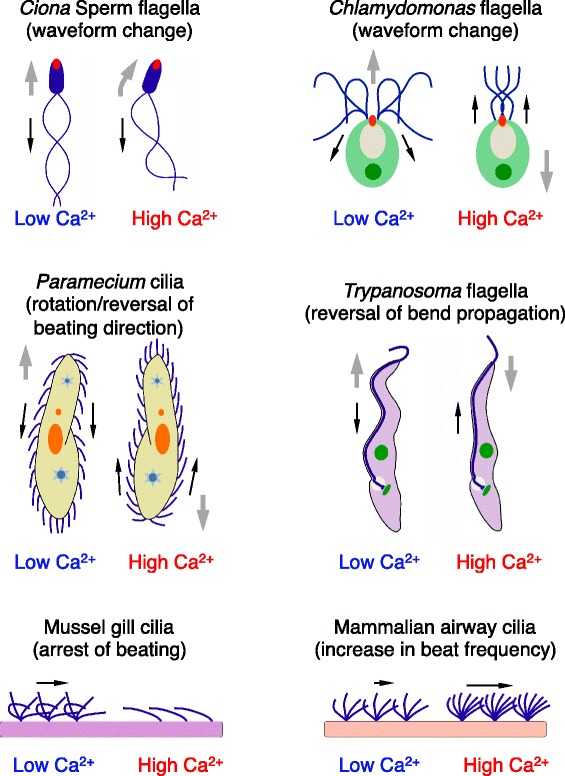
**Schematic drawings of various Ca** ^**2+**^ **-dependent changes in wave propagation of cilia and flagella and the direction of locomotion and water flow in several organisms and tissues.** Red dots in *Ciona* sperm and *Chlamydomonas* flagella indicate acrosomes and mating structure (fertilization tubules), respectively. Black and gray arrows represent the direction of wave propagation and cell locomotion, respectively.

Cilia and flagella respond to extracellular stimuli and change their motility. Ca^2+^ is a well-known intracellular regulator for modulation of ciliary and flagellar movements. These modulations range across diverse modes, including (1) changes in ciliary or flagellar waveforms, (2) rotation or reversal of the direction of ciliary or flagellar bending, (3) arrest of beating, and (4) increasing of beat frequency (Figure 1).

#### Changes in ciliary or flagellar waveforms

Sperm swim with the tip of the head (acrosome) ahead of the direction of movement for fertilization of the egg. Sperm of the ascidian *Ciona intestinalis* dramatically increase flagellar asymmetry in response to increases in intracellular Ca^2+^ concentration caused by a chemoattractant from the egg [9,10]. This change enables the sperm to make turns and move forward towards the egg.

The unicellular alga *Chlamydomonas reinhardtii* has two flagella and usually swims in breast stroke fashion with the flagella located anterior to the cell body. A structure for mating is formed between the two flagella at fertilization [11,12]. When exposed to intense light, *Chlamydomonas* stops its motility and then moves in the reverse direction with conversion of flagella into a symmetrical waveform [13]. Analysis with a demembranated cell model suggests that the conversion of flagellar waveform from asymmetric to symmetric is caused by an increase in Ca^2+^ concentration. The increase of intracellular Ca^2+^ appears to be performed by Ca^2+^ influx through a voltage-dependent channel CAV2 [14]. Similar flagellar response to Ca^2+^ is observed in the prasinophyte *Spermatozopsis similis* [15].

#### Rotation or reversal of the direction of ciliary or flagellar bending

In *Paramecium*, Ca^2+^ causes reversal of the beating plane of cilia [16-18]. Extracellular stimuli such as mechanical collision induce membrane depolarization and subsequent Ca^2+^ influx, resulting in ciliary reversal and backward swimming. It is considered that ciliary reversal in *Paramecium* cilia is caused by the rotation of the central pair in the axoneme [19,20]. Rotation through 180° causes complete reversal of the beating plane of the cilia. In the case of Ctenophora, the ciliary comb plate also shows ciliary reversal in a Ca^2+^-dependent manner [21]. However, these comb plate cilia perform reversal of the beating plane without rotation of the central axoneme pair [22].

*Trypanosoma* propagate flagellar waves both from the base to tip and the tip to base [23,24]. Demembranated cell models demonstrate that the direction of flagellar bend propagation reverses when the cell is demembranated by glycerol or detergent and is reactivated by ATP at low concentrations of Ca^2+^ in the trypanosomatid *Crithidia oncopelti* [25].

Sperm in some insects and snails reverse the direction of bend propagation in a Ca^2+^-dependent manner [26-30]. For example, in the sperm of the gastropod *Strombus luhuanus*, the reversal of bend propagation appears to be involved in sperm release from the sperm storage site in the female genital tract [30].

#### Arrest of beating

Epithelial cilia of marine invertebrates show ciliary arrest in response to Ca^2+^. Spontaneous arrest of mussel gill cilia is caused by membrane depolarization, depending on calcium ions [31,32]. Most of the gill cilia in demembranated cell models show arrest of beating at >10^−4^ M Ca^2+^ [33]. Ciliary arrest in *Ciona* stigmatal cells also depends on the presence of external Ca^2+^ [34]. Cilia of sea urchin embryos or larvae undergo a series of changes in the beating pattern. Spontaneous ciliary arrest is observed in early stages of development; in later stages, cilia show spontaneous reversal or arrest and increase in beat frequency. In many cases, these changes are accelerated by the presence of the Ca^2+^ ionophore A23187 in seawater [35].

#### Increase in beat frequency

Ca^2+^ induces increased beat frequency in airway cilia in mammals [36-38] and in oviductal cilia [39], without alteration of beating direction. Increase in beat frequency is also observed in the Triton-extracted *Paramecium* model and is inhibited by a calmodulin (CaM) antagonist [18]. However, sperm flagella show no significant increase in beat frequency due to Ca^2+^ in sea urchin [2] or *Ciona* (Mizuno and Inaba, unpublished observation), although a demembranated model of sea urchin sperm flagella changed to an asymmetric waveform on stimulation with Ca^2+^, and showed quiescence at Ca^2+^ concentration >10^−4^ M [40].

The effects of Ca^2+^ on ciliary and flagellar motility appear diverse among organisms, but the roles of Ca^2+^ in the regulation can be classified into two parts. One is a signaling pathway upstream of the modulation of the axonemes. Influx of Ca^2+^ is an important trigger for the modulation of ciliary and flagellar motility. Several Ca^2+^ channels and Ca^2+^-binding enzymes, such as protein kinases and phosphatases, have been reported to be localized and functional in the ciliary/flagellar plasma membrane and the ciliary/flagellar matrix [6,7]. The other is the direct modulation of axonemal movements. Ca^2+^-binding proteins such as calaxin, dynein light chain 4 (LC4), CaM, and centrin are bound to the substructures of the axonemes and directly modulate dyneins or their regulatory elements, the radial spokes, and central apparatus. In this paper, I focus on the Ca^2+^ sensors that directly act on the outer arm dynein in the axonemes.

### Outer arm dynein is essential for Ca^2+^-mediated changes of ciliary movement

The extent of flagellar or ciliary bending correlates with the velocity of microtubule sliding [41,42]. The flagellar waveform is composed of a bend with a larger angle (principal bend) and an opposite bend with a smaller angle (reverse bend) [2]. Formation of bends and propagation are achieved by local microtubule sliding, for which dyneins are considered to be locally activated on one side to bend the axoneme, while those on the other side are inactive [2,43].

The central apparatus (CP) - along with the radial spokes (RS) - plays an important role in flagellar motility as revealed by the paralysis of *Chlamydomonas* CP mutants [44,45]. The CP is involved in determining the bending plane, demonstrated by the helical movement with 9+0 axonemal structures of eel and Asian horseshoe crab sperm [46,47], and the loss of planar bend movement and the development of helical movement after treatment of a sperm model by antibodies against radial spokes [48]. The activation of specific axonemal dyneins by CP/RS is thought to enable mutual sliding of microtubules across the axoneme, resulting in planar bend propagation [49-51]. Studies on *Chlamydomonas* flagella have shown that signals from the central apparatus activate specific dyneins for local bending [45,52]. As previously reported, the f (I1) inner arm dynein is regulated by phosphorylation/dephosphorylation of a 138 kDa intermediate chain (IC) through a kinase/phosphatase system present in the RS and CP [53,54].

Axonemes have two dynein motors with different properties: outer arm dynein and inner arm dynein. Subunits of the outer arm dynein have been well studied in *Chlamydomonas* and in the sperm of *Ciona* and sea urchins [7,55-59]. They have two or three motor subunits (heavy chains) in the sperm or *Chlamydomonas*, respectively. Other subunits, including intermediate chains and light chains, are involved in the assembly and regulation of dyneins. Several studies with *Chlamydomonas* mutants and outer arm extracted sea urchin sperm indicate that outer and inner arm dyneins are involved in the elevation of microtubule sliding velocity (increasing beat frequency) and formation and propagation of flagellar bending, respectively [3,4].

Much experimental evidence demonstrates that outer arm dynein is essential for Ca^2+^-dependent modulation of ciliary motility. The conversion of flagellar wavelength from symmetric to asymmetric is transiently observed during chemotaxis of the sperm to the egg [9,60,61]. This is caused by Ca^2+^-dependent regulation of outer arm dynein (see below). Lack of outer arm dynein in the human sperm causes low swimming velocity, loss of circular movement with an asymmetric waveform, and low efficiency of penetration into the egg coat [62,63].

*Chlamydomonas* changes swimming direction in response to light. There are two types of response: a photophobic reaction to very strong light, photoshock, and a positively or negatively directed movement towards a light source, phototaxis. Both photoshock and phototaxis depend on changes in intracellular Ca^2+^. Reactivated *Chlamydomonas* axonemes show an asymmetric beat pattern at Ca^2+^ concentrations below 10^−6^ M, become quiescent at 10^−5^ M, and then resume beating with a symmetric waveform at 10^−4^ M [64]. This waveform conversion does not occur in mutants lacking dynein outer arms [58,59,65]. In contrast, phototaxis is caused by different responses of the cis- and trans-flagellum. The cis- and trans- flagellar axonemes of demembranated *Chlamydomonas* cell models differentially respond to Ca^2+^ concentration in the range 10^−8^ M to 10^−6^ M [57]. Studies using axonemal dynein mutants indicate that phototaxis requires the inner, but not the outer, row of dynein arms [58,59].

Specific knockdown of outer arm dynein LC1 in *Trypanosoma brucei* results in the loss of tip to base propulsive propagation of the flagellar wave [66] that is usually observed in normal forward swimming. A similar phenotype is obtained when LC2 is knocked down [67]. The tip to base propagation is Ca^2+^-dependent, and the base to tip propagation is only observed in demembranated models when demembranated and reactivated in the presence of EGTA [25]. RNAi knockdown of LC1 in the planarian *Schmidtea mediterranea* demonstrated that outer arm dynein is essential for the increase of beat frequency and coordination of cilia to produce ciliary oscillation with metachronal waves [68].

### Calaxin is the calcium sensor of outer arm dynein necessary for chemotactic turns of the sperm with asymmetric waveforms

**Figure 2:**
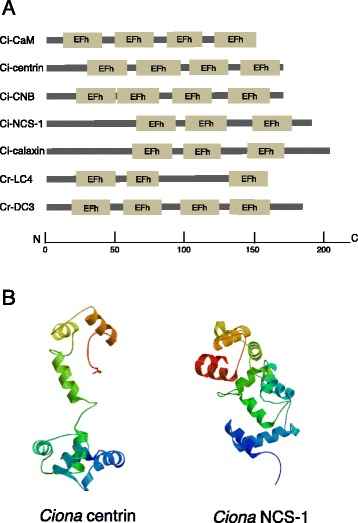
**Structures of EF-hand Ca** ^**2+**^ **-binding proteins. (A)** Domain structures of *Ciona* and *Chlamydomonas* Ca^2+^-sensors, drawn based on SMART searches (http://smart.embl-heidelberg.de/). The length of each protein and the positions of EF hand motifs are scaled below. **(B)** Molecular models of ligand-unbound *Ciona* centrin and NCS-1, built using SWISS-MODEL (http://swissmodel.expasy.org) [175]. Templates used are 1tnx.1 (skeletal muscle troponin) and 2d8n.1 (human recoverin) for *Ciona* centrin and NCS-1, respectively.

Changes in ciliary and flagellar motility by Ca^2+^ are mediated by Ca^2+^-binding proteins. The most common motif for Ca^2+^ binding is the EF hand. It is a helix-loop-helix structural motif of 12 residues (+X)x(+Y)x(+Z)x(−Y)x(−X)xx(−Z) for metal coordination, where +X, +Y, +Z and −X, −Y, −Z are the vertices of an octahedron [69-71]. The EF hand family contains the CTER, CRP, and S100 subfamilies. These three show mutual congruence to one another within a subfamily. There are many other subfamilies containing EF hands with no strong congruence to one another (Table 1) [72]. Both CTER and CRP basically contain four EF hands, at least one of which lacks the capacity to bind Ca^2+^ in CRP and does not match the consensus sequence in a PROSITE search (Figure 2A). CTER subfamily proteins, such as CaM, centrin, and troponin C, have dumbbell shape structures with two globular lobes connected by an eight-turn α-helix, whereas CRP, such as recoverin and NCS-1 (frequenin), have a globular structure without the long α-helix link (Figure 2B) [73].

**Table 1:** **Classification of EF-hand Proteins**

**Family**	**Subfamily**	**Proteins**
**CTER (calmodulin, troponin C, essential and regulatory light chain) subfamily**		calmodulin, troponin C, essential light chain of myosin, regulatory light chain of myosin
		Others: Ca^2+^/CaM protein kinase, calsenilin, DREAM, calcineurin B like (CBL) sensors
**CRP (calcineurin B, p22, recoverin) subfamily**		calcineurin B, p22, recoverin (and other neuronal calcium sensor family)
		Others: Ca^2+^/CaM protein kinase, calsenilin, DREAM, calcineurin B like (CBL) sensors
**S100 subfamily**		S100, calbindin D_9k_, P26olf (dicalcin)
		Fused gene family: profilaggrin, trichohyalin, repetin, hornerin, profilaggrin-related protein, cornulin
**Other EF-hand subfamilies showing no strong congruence to one another**	PENTA-EF-hand subfamily	Group II (calpain, calcium-dependent proteases, sorcin, grancalcin), Group I (ALG-2, peflin)
	Proteins with six EF-hands	calbindin D28k, calretinin, Eps15 homology domain, CREC family (Ca^2+^-binding protein of 45 kDa, reticulocalbin, ER Ca^2+^-binding protein of 55 kDa, and calumenin), *Plasmodium falciparum* surface protein, calsymin
	Proteins with eight and 12 EF-hands	*Lytechinus pictus* SPEC-resembling protein, EF12
	Proteins with four EF-hands	*Tetrahymena pyriformis* calcium binding protein TCBP-25, TCBP-23, CBP, calcyphosine, *Strongyrocentrotus purpuratus* Ectoderm calcium binding protein SPEC, calflagin, sarcoplasm calcium binding protein, *Hra32*, EFH5, calcium vector protein, PM129 clone from Arabidopsis thaliana, calcium and integrin binding protein, spasmin, aequorin, *Plasmodium falciparum* protein kinase (PFCPK), protein phosphatase encoded by *rdgC*, phospholipase C, LAV1
	Proteins with two-EF-hands	Phl p 7 (polcalcin), alpha-actinin, alpha-spectrin, calmodulin-related gene product encoded by T+, calsensin, groovin, diacylglycerol kinase, glycerol-3-phosphate dehydrogenase, fimbrin, ras guanyl nucleotide-releasing protein (GRP), polycystin-2, ryanodine receptor protein (RYR), CBL, nucleobindin, allograft inflammatory factor-1, BM-40 (osteonectin)
	EF-hand proteins in bacteria and viruses	calerythrin, other EF-hand proteins in bacteria, MSV from pox virus

The list is based on the chapter 11 of a book by Permyakov and Kretsinger (2011) [69].

Many studies showed that CaM is an important Ca^2+^ sensor for the regulation of ciliary and flagellar movements [74,75]. Although CaM was a strong candidate to be the Ca^2+^-dependent regulator for outer arm dynein in the sperm, several experiments suggest the presence of Ca^2+^-binding proteins other than CaM. Unlike the light chain 4 (LC4) in *Chlamydomonas*, the outer dynein could not be isolated from sperm flagella in association with any Ca^2+^-binding proteins. Moreover, conversion into an asymmetric flagellar waveform is achieved at high concentrations of Ca^2+^ in the sea urchin sperm model demembranated by Triton X-100 in the presence of millimolar Ca^2+^ [2,40]. In this condition, CaM is extracted from the axonemes. These reactivated sperm models called ‘potentially symmetric’ sperm show symmetric waveforms at low concentrations of Ca^2+^ but become asymmetric when Ca^2+^ is increased in the reactivation medium. The asymmetric flagellar waveform is seen only in the presence of high concentrations of ATP [40], which induces motility with high beat frequency and therefore implies a role of outer arm dynein.

Ca^2+^-dependent conversion of flagellar waveform is essential for sperm chemotaxis [9,10,60,76-79] and rheotaxis [80], response of sea urchin sperm to mechanical stimuli [81], self-nonself recognition of sperm [82], hyperactivation [83,84], and release from the epithelium of sperm storage sites [85,86]. In the ascidian *Ciona intestinalis*, correlation between the increase in intracellular Ca^2+^ concentration and conversion of flagellar asymmetry is clearly observed [9]. *Ciona* sperm show rather planar wave propagation in seawater with a slight asymmetric flagellar waveform, resulting in a circular trajectory. The reception of the gradient of chemoattractant (sperm activating and attracting factor; SAAF) from the egg [87] induced a transient increase in intracellular Ca^2+^ concentration. Flagellar axonemes respond to the change and temporarily form and propagate an asymmetric waveform, resulting in a turning movement towards the egg [9].

**Figure 3:**
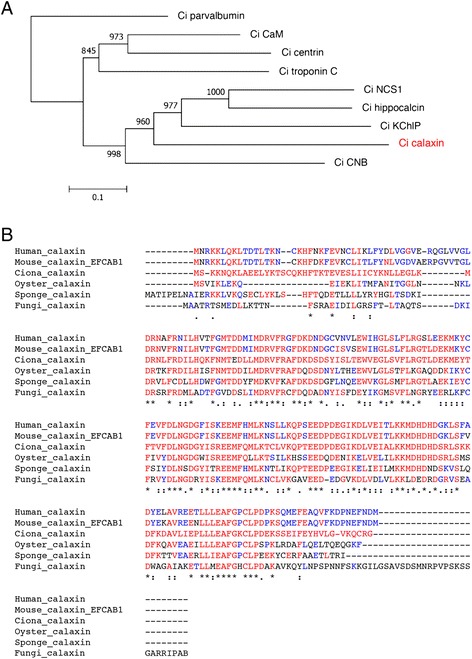
**Calaxin is an opisthokont-specific Ca** ^**2+**^ **sensor. (A)** A phylogenetic tree of Ca^2+^-binding proteins in the ascidian *Ciona intestinalis*. Proteins were aligned by CLUSTALW, and the tree was constructed by MEGA5. *Ciona* parvalbumin-like protein (XP_002129217) was used as the outgroup. The value shown on each branch represents the number of times that a node was supported in 1,000 bootstrap pseudo-replications. Accession numbers or NCBI reference sequence numbers of the sequence resources are as follows: calmodulin (AB076905), calaxin (AB079059), centrin (XP_004227465), troponin C (XP_002129347), NCS-1 (XP_002126443), hippocalcin (XP_002124848), KChIP (XP_004226075), calcineurin B subunit (CNB) (XP_002130765). **(B)** Multiple alignment of calaxin in opisthokont species. Asterisks, colons, or dots indicate identical residues in all sequences in the alignment, conserved substitutions, or semi-conserved substitutions, respectively. The amino acid residues identical to *Ciona* calaxin or to calaxin in other organisms are in red or blue, respectively. The sources of amino acid sequences are as follows: human calaxin (NP_078869), mouse calaxin (NP_080045), *Ciona* calaxin (AB079059), oyster calaxin (EKC38288), sponge calaxin (XP_003383675), and chytrid fungus calaxin (XP_006677085).

A previous study found a Ca^2+^-binding protein that is expressed in *Ciona* testis during the course of extensive descriptions of axonemal proteins [88]. It turned out that this protein is an axonemal protein localized at the outer arm dynein, named Ca^2+^-binding axonemal protein calaxin [89]. Calaxin is grouped into one of the CRP EF hand protein families, the neuronal calcium sensor (NCS) protein family, which is expressed in retinal photoreceptors or neurons and neuroendocrine cells [90,91]. A phylogenetic analysis shows that calaxin is a new type of NCS protein in the axoneme; other proteins, such as CaM and centrin, are all grouped into different phylogenetic clades (Figure 3A).

Calaxin has three Ca^2+^-binding EF hand motifs (amino acids 62 to 90, 98 to 126, and 151 to 166 in *Ciona* calaxin) [10,89]. Ca^2+^-binding to these sites was directly demonstrated by isothermal titration calorimetry (ITC), showing a three-site sequential binding model [10]. Two of the three EF hand motifs exhibited endothermic binding and the other exothermic binding. Ca^2+^-dependent hydrophobic interactions are suggested from positive enthalpy in ITC, as in the case of Ca^2+^ binding to calmodulin [92]. Several investigations demonstrate membrane-associated roles of NCS in the modulation of neurotransmitter release, biosynthesis of polyphosphoinositides, and in the direct regulation of ion channels [93,94]. In fact, the N-termini of NCS proteins are myristoylated and become exposed outside the protein molecules by binding of Ca^2+^, allowing them to associate with membranes. The consensus sequence for myristoylation, N-terminal GXXXSX [94], is found in mammalian NCS-1 and calcineurin B. However, it is not present in calaxin or its mammalian orthologs [89], suggesting that the N-terminal is not myristoylated and that calaxin does not have the Ca^2+^-myristoyl switch property of NCS. Immunohistochemical observations indicate that calaxin is located on the outer arm dyneins along the axoneme of sperm flagella [89]. Calaxin is also distributed in the cilia of ciliated tissues, such as the branchial basket and endostyle [84]. Far western blotting shows that calaxin binds to β-tubulin in the absence of Ca^2+^ and to the β heavy chain (ortholog of the *Chlamydomonas* γ heavy chain) of the outer arm dynein [89]^a^. Calaxin binds to the N-terminal stem region, as revealed by far-western blotting against UV-cleaved fragments of the β heavy chain (Mizuno and Inaba, unpublished data). Although two IQ consensus motifs for binding CaM-like proteins are located within the stem domain of the *Chlamydomonas* γ heavy chain [95], there is no such motif in the corresponding region of the *Ciona* β heavy chain.

*Ciona* sperm shows a unique turning movement associated with a flagellar change to asymmetric waveforms, followed by straight-ahead movement towards the chemoattractant SAAF [87]. In the presence of an NCS inhibitor, repaglinide, the sperm do not exhibit this unique turning movement, showing less-effective chemotaxis [10]. Repaglinide-treated sperm can transiently form asymmetric flagellar waveforms in the gradient of chemoattractant. However, they do not sustain the asymmetric waveform and rapidly return to a symmetric form, resulting in less chemotactic behavior. The flagellar waveforms of sperm demembranated with 0.04% Triton X-100 become more asymmetric when reactivated at >10^−6^ M Ca^2+^. Repaglinide attenuates propagation of asymmetric waveforms, but not the relatively symmetric waveforms seen at low concentrations of Ca^2+^. Calaxin directly suppresses the velocity of microtubule sliding by outer arm dynein at high Ca^2+^ concentrations. Repaglinide and anti-calaxin antibody cancel the suppression of microtubule translocation at high concentrations of Ca^2+^. All of these data demonstrate that calaxin plays an essential role in the propagation of asymmetric flagellar bending by suppression of dynein-driven microtubule sliding at high concentration of Ca^2+^ [10]. Calaxin appears evenly located to every doublet microtubule [89]. Then, how does calaxin work to propagate an asymmetric planar waveform, in which dyneins on the two sides of axonemes mainly participate in microtubule sliding? Although there has not been any experimental evidence to elucidate this question, the function of calaxin might be regulated through a mechanical feedback, such as thrust from flagellar bending, or through a biochemical mechanism, such as protein phosphorylation and dephosphorylation.

BLASTP searches for *Ciona* calaxin in the genomes of *Chlamydomonas reinhardtii* and *Paramecium tetraurelia* hit hypothetical proteins CHLREDRAFT_119565 (XP_001696107) (E = 4e^−13^) and XP_001433234 (E = 2e^−15^), respectively. Both hypothetical proteins show a best match with calcineurin subunit B type 1-like protein (CBL-1), not calaxin, in the *Ciona* genome. LC4 is a Ca^2+^-binding subunit of outer arm dynein first identified in *Chlamydomonas* [96]. It shows sequence similarity to CaM and CaM-related proteins such as centrin/caltractin and troponin C. Ca^2+^-binding assays demonstrate that LC4 has at least one functional Ca^2+^-binding site. LC4 is isolated in association with the γ heavy chain of outer arm dynein. These properties suggest functions of LC4 analogous to those of calaxin, although the proteins are phylogenetically distinct from each other.

### Calaxin is an opisthokont-innovated calcium sensor in cilia and flagella

The current view of eukaryote phylogeny includes its basal division into unikonts (Opisthokonts and Amoebozoa) and bikonts (Archaeplastida, Hacrobia, Stramenopiles, Alveolates, Rhizaria, and Excavata), based on the concept of eukaryotic cells with a single flagellum or two flagella, respectively. Opisthokonts are groups shown to propel cells by a posterior flagellum [97-99]. Homologs of calaxin were searched in available genome databases. Calaxin homologs were not found in any bikont species, such as Archaeplastida (*Chlamydomonas*) or Stramenopiles (ciliates, dinoflagellates, and blown algae). Calaxin homologs were only found, and were well conserved, in species of the opisthokont supergroup, including *Homo sapiens*, *Mus musculus*, *Ciona intestinalis*, *Strongylocentrotus purpuratus*, *Amphimedon queenslandica*, *Drosophila melanogaster*, *Monosiga brevicollis*, and *Crassostrea gigas*. The opisthokont organisms that lack motile cilia or flagella throughout their life cycles, such as *C. elegans*, Vericrustaceans (except Notostraca and Thecostraca), yeast, and higher fungi show no calaxin gene in their genomes, although these organisms have genes for other NCSs such as NCS-1 (frequenin). The chytrid fungus *Batrachochytrium dendrobatidis*, grouped into the opisthokonta with metazoa, contains a calaxin gene (XP_006677085) in its genome. The calaxin of *B. dendrobatidis* shares 38% amino acid identity with *Ciona* calaxin (Figure 3B). Because of insufficient genome information, the presence of calaxin in Amoebozoa has not been elucidated. BLASTP searches show that calaxin is not present in either the aflagellate amoebozoan *Dictyostelium discoideum* or the flagellated amoebozoid *Breviata anathema* which lacks outer arm dynein [100]. However, one of the well-investigated genera in the Amoebozoa, *Physarum polycephalum*, has a flagellated period in its life cycle. Because it possesses an axoneme of 9+2 structure with outer arm dynein [101-103], it is possible that calaxin could be present in Amoebozoa and could be a unikont-innovated protein.

A previous study identified proteins with a unique combination of domains: an intermediate chain of outer arm dynein, thioredoxin domain and nucleoside diphosphate kinase domain (TNDK-IC, [104,105]) and a radial spoke protein CMUB116 (IQ motif and ubiquitin domain [106]). These proteins are also opisthokont-specific proteins, suggesting that a critical evolutionary event occurred during specification of axonemes in the opisthokont lineage.

### Mirror-image relationship between calaxin and LC4

Knowledge of the molecular components of axonemal dyneins and the molecular mechanism of ciliary and flagellar motility has been accumulated mostly from metazoan sperm and certain protists such as *Chlamydomonas*. In the present study, an attempt has been made to biochemically compare the outer arm dynein and its Ca^2+^ sensor between *Ciona* sperm flagella and *Chlamydomonas* flagella and to correlate their functions in the regulation of motility.

The outer arm dynein of *Ciona* sperm flagella consists of two heavy chains and represents a two-headed structure, but that of *Chlamydomonas* flagella consists of three heavy chains with a three-headed structure. Each of the two heavy chains of sperm outer arm dynein is known to have distinct properties [107-110]. Sea urchin α heavy chain (an ortholog of *Ciona* β and *Chlamydomonas* γ heavy chains) mediates structural and rigor binding to the microtubules [110]. *In vitro* motility assays indicate that the absence of the *Chlamydomonas* γ heavy chain increases both microtubule gliding and ATPase activity [111], suggesting that the γ heavy chain suppresses the activities of outer arm dynein.

**Figure 4:**
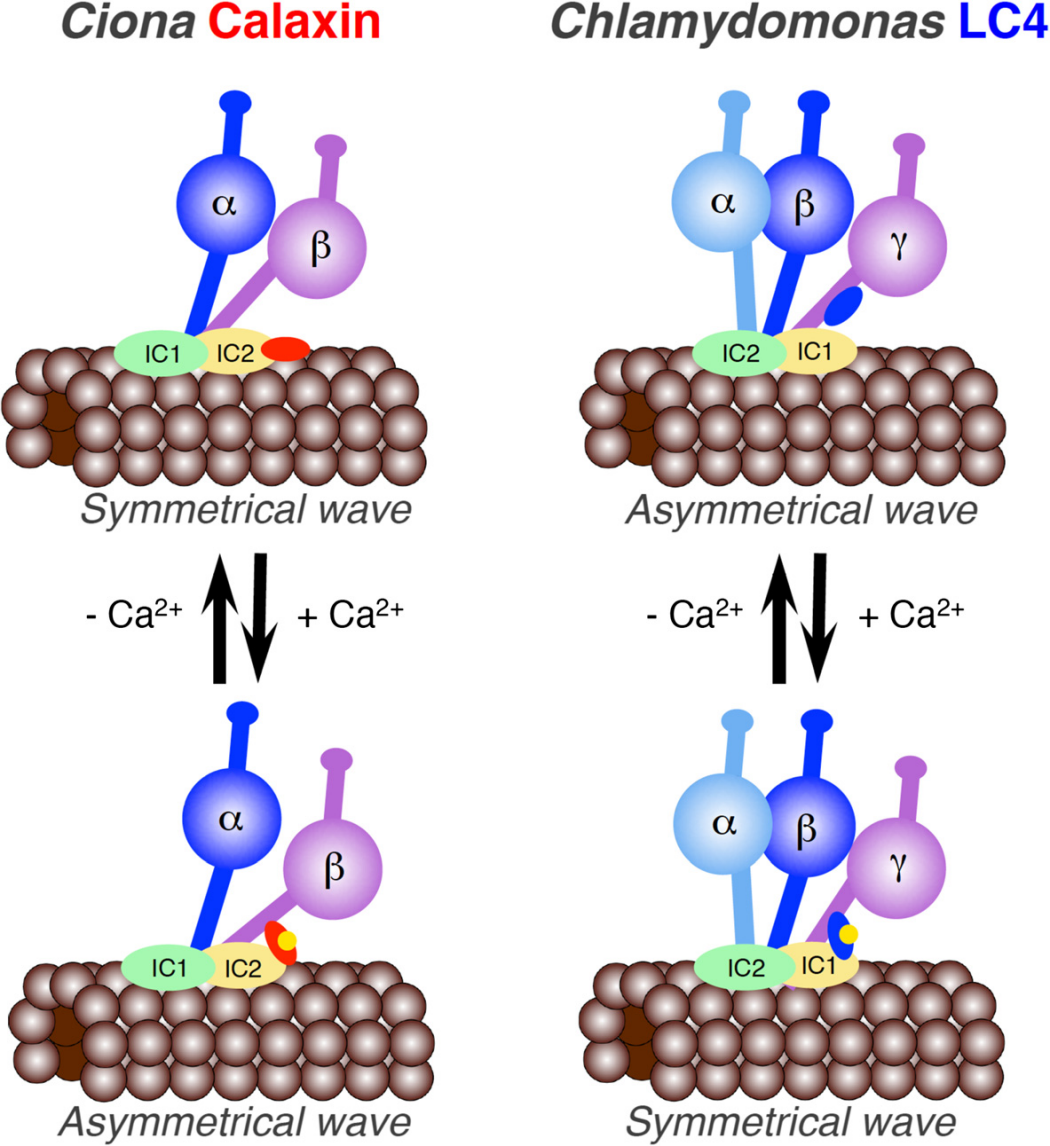
**Mirror image in the function of outer arm dynein Ca** ^**2+**^ **sensors between** ***Ciona*** **and** ***Chlamydomonas*** **.** *Ciona* calaxin binds to the β-heavy chain, suppresses microtubule-sliding and induces propagation of an asymmetric waveform at high concentration of Ca^2+^. In contrast, *Chlamydomonas* LC4 binds to the γ-heavy chain, becomes tethered to IC1 and induces propagation of a symmetric waveform at high concentration of Ca^2+^. Direct evidence for the activation of microtubule-sliding by *Chlamydomonas* outer arm dynein has not been obtained.

*Ciona* calaxin and *Chlamydomonas* LC4 bind to *Ciona* β and *Chlamydomonas* γ heavy chains, respectively [89,112]. However, Ca^2+^ dependency of the binding is reversed between *Ciona* and *Chlamydomonas* (Figure 4). Calaxin binds to intermediate chain 2 (IC2) and β tubulin in the absence of Ca^2+^ but becomes associated with the β heavy chain at higher concentrations of Ca^2+^ [89]. The binding of calaxin to the heavy chain results in the suppression of microtubule-gliding activity by outer arm dynein [10]. In the case of *Chlamydomonas*, LC4 is bound to the γ heavy chain in the absence of Ca^2+^ but becomes newly tethered to IC1 (an ortholog of *Ciona* IC2) in the presence of Ca^2+^ [95,112]. Although the effect of Ca^2+^ binding to LC4 on dynein-driven microtubule sliding has not been examined in *Chlamydomonas*, the binding of Ca^2+^ to LC4 induces activation of ATPase activity of the outer arm dynein in the mutant lacking the α heavy chain [112]. A model has been proposed for Ca^2+^-dependent regulation of the γ heavy chain; in the absence of Ca^2+^, LC4 is tightly bound to the γ HC, resulting in inefficient formation of a rigor bond with microtubules. In the presence of high Ca^2+^, Ca^2+^-bound LC4 detaches from the IQ region of the γ heavy chain and becomes attached to IC1, resulting in a structural change of the N-terminal stem domain and the activation of motor activity [95].

These mirror-image relationships in the effect of Ca^2+^ on the regulation of outer arm dynein in *Ciona* and *Chlamydomonas* are likely to connect with the difference in the changes of flagellar waveforms (Table 2). At high concentrations of intracellular Ca^2+^, *Ciona* sperm show asymmetric waveforms whereas *Chlamydomonas* flagella become symmetric. The molecular mechanisms of Ca^2+^-dependent regulation of outer arm dynein appear quite similar to each other, but the response to Ca^2+^ in the conversion of flagellar waveforms is completely reversed. This implies the possibility of an evolutionary event in the functional diversification of cilia and flagella at the onset of eukaryotic radiation.

**Table 2:** **Comparison of Ca** ^**2+**^ **-dependent regulation of outer arm dynein between** ***Ciona*** **sperm flagella and** ***Chlamydomonas*** **flagella**

	***Ciona***	***Chlamydomonas***
Outer arm dynein	Two-headed	Tree-headed
Ca^2+^sensor	Calaxin	LC4 (+DC3)
Target heavy chain	β HC^a^	γ HC^a^
Low Ca^2+^	Binding to IC2^b^	Binding to HC
	Binding to β-tubulin	
High Ca^2+^	Binding to HC	Binding to HC
	Binding to IC2	Binding to IC1^b^
MTs sliding	Suppression	ND
Flagellar asymmetry	High Ca^2+^	Low Ca^2+^

The nomenclatures of the components of dynein are confusing because these were originally named based on electrophoretic mobility in SDS-gel (see references [56,57,166,174]). ^a^ *Chlamydomonas* γ HC corresponds to *Ciona* β HC. ^b^ *Chlamydomonas* IC1 corresponds to *Ciona* IC2.

It is unlikely that ciliary response in waveform conversion depends on the extracellular Ca^2+^ concentration in the environment (such as in seawater or freshwater). For example, sperm of freshwater fish show asymmetric waveforms depending on an increase in intracellular Ca^2+^ concentration [113,114]. The marine alga *Pyramimonas parkae* shows waveform conversion similar to *Chlamydomonas reinhardtii* [115], although the relationship between the conversion and intracellular Ca^2+^ concentration has not been elucidated. An interesting experiment was the examination of the relationship between intracellular Ca^2+^ concentration and flagellar waveform in the prasinophyte algae *Pterosperma* and *Cymbomonas*, both of which show conversion of flagellar waveforms similar to metazoan sperm: symmetric flagellar waveforms in normal swimming and asymmetric waveforms when they change swimming direction [115]. Anterior flagella of Stramenopiles bear hair-like structures called mastigonemes [116]. These organisms or their gametes normally swim with the anterior flagellum ahead. The flagella show symmetric wave propagation from base to tip, but the direction of propulsive force changes because of the reversal of water current by mastigonemes [117]. They change swimming direction in phototactic behavior by altering the flagellar waveform or the orientation of the anterior or posterior flagellum [118], but the relationship between waveform change and intracellular Ca^2+^ is unclear.

### Usage of distinct Ca^2+^ sensors in unikont and bikont supergroups

**Figure 5:**
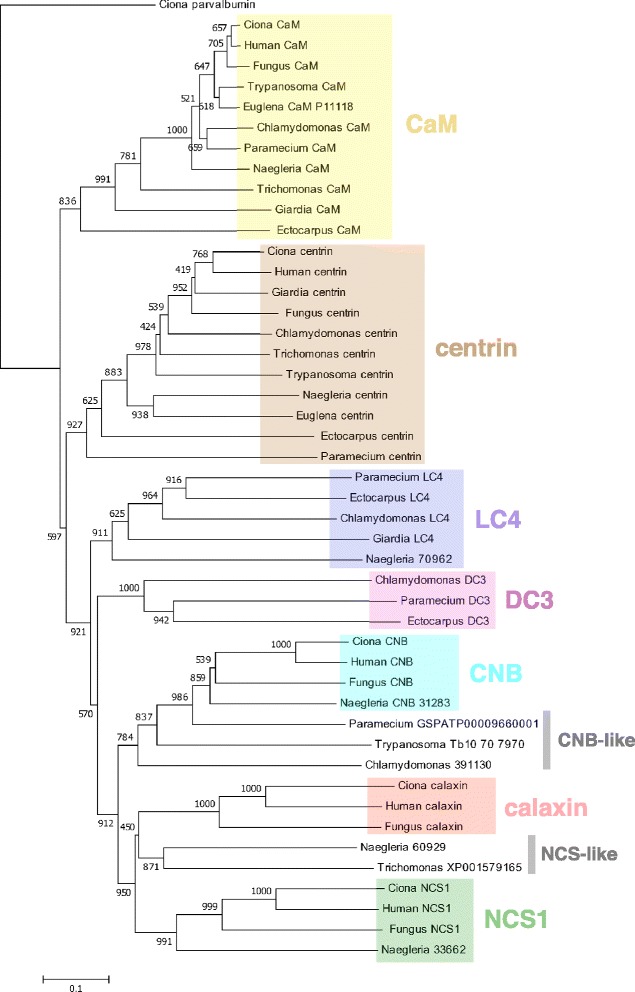
**Phylogenetic analysis of Ca** ^**2+**^ **-binding proteins.** Proteins were aligned by CLUSTALW, and the tree was constructed by MEGA5. *Ciona* parvalbumin-like protein (XP_002129217) was used as the outgroup. The value shown on each branch represents the number of times that a node was supported in 1,000 bootstrap pseudo-replications. Sequences were obtained from the organisms *Ciona* (*Ciona intestinalis*), human (*Homo sapiens*), fungus (*Batrachochytrium dendrobatidis*), *Naegleria* (*Naegleria gruberi*), *Euglena* (*Euglena gracilis*), *Trypanosoma* (*Trypanosoma cruzi* or *T. brucei*), *Giardia* (*Giardia intestinalis* or *G. lamblia*), *Trichomonas* (*Trichomonas vaginalis*), *Chlamydomonas* (*Chlamydomonas reinhardtii*), *Paramecium* (*Paramecium tetraurelia*), and *Ectocarpus* (*Ectocarpus siliculosus*). The sources of amino acid sequences are as follows: *Ciona* calmodulin (AB076905), *Ciona* calaxin (AB079059), *Ciona* centrin (XP_004227465), *Ciona* NCS-1 (XP_002126443), *Ciona* CNB (XP_002130765); human CaM (CAA36839), human calaxin (NP_078869), human NCS1 (NP_055101), human CNB (NP_000936), human centrin (NP_004057); chytrid fungus calaxin (XP_006677085), chytrid fungus CaM (XP_006678916), chytrid fungus centrin (XP_006682970), chytrid fungus NCS1 (XP_006675998), chytrid fungus CNB (XP_006677028); *Naegleria* CaM (XP_002683533), *Naegleria* centrin (XP_002678269); *Trypanosoma* CaM (XP_805243), *Trypanosoma* centrin (XP_805423), *Trypanosoma* calflagin (Q26680); *Euglena* CaM (P11118), *Euglena* centrin (AGS09408); *Giardia* CaM (XP_001705820), *Giardia* centrin (XP_001707577), *Giardia* LC4 (XP_001705117); *Trichomonas* CaM (XP_001326924), *Trichomonas* centrin (CAB55607), *Trichomonas* CNB (XP_002680632); *Paramecium* CaM (XP_001448363), *Paramecium* LC4 (XP_001442002), *Paramecium* centrin (XP_001347281), *Paramecium* DC3 (XP_001444482); *Ectocarpus* LC4 (CBN80105), *Ectocarpus* CaM (CBN74265), *Ectocarpus* centrin (CBN79657), *Ectocarpus* DC3 (CBJ30770). The protein sequences with specific accession numbers were obtained from DDBJ/EMBL/GenBank, or from genome browsers with the following URLs: *Chlamydomonas* http://genome.jgi-psf.org/Chlre4/Chlre4.home.html; *Paramecium* http://paramecium.cgm.cnrs-gif.fr; *Naegleria* http://genome.jgi-psf.org/Naegr1/Naegr1.home.html; *Trichomonas* http://trichdb.org; and *Trypanosoma* https://www.sanger.ac.uk/resources/downloads/protozoa/trypanosoma-brucei.html.

A phylogenetic analysis of *Ciona* calaxin, CaM, centrin, NCS, calcineurin B-subunit (CN-B), *Chlamydomonas* LC4, and a Ca^2+^-binding subunit of outer arm dynein docking complex 3 (DC3) [119,120] using available genome information resulted in distinct distribution of calaxin and LC4/DC3 in the opisthokont and bikont supergroups, respectively (Figure 5). *Chlamydomonas* LC4 and its orthologs were grouped into a clade different from that of calaxin but were more closely related to calaxin than were CaM or centrin. BLASTP searches of *Chlamydomonas* LC4 against genomes of bikonts resulted in finding orthologs in flagellated species including ciliates, dinoflagellates, diatoms, brown algae, haptophytes, and cryptophytes. Exceptions are seen in organisms lacking outer arm dynein such as angiosperm, moss, and fern [121]. BLASTP searches of *Chlamydomonas* LC4 against these species resulted in best hits to CaM. Search of *Chlamydomonas* LC4 in the genomes of opisthokonts failed to find any homologs in this supergroup. For example, the protein most homologous to LC4 in *Ciona intestinalis* was CaM (E = 3e^−22^).

DC3 is also a CaM type of EF hand protein localized on the outer arm dynein docking complex and shows redox-sensitive Ca^2+^-binding with a ratio of 1 mol Ca^2+^/mol protein [120]. However, it is unclear whether DC3 actually binds Ca^2+^ under physiological conditions because it also significantly binds Mg^2+^ [122]. Genes of DC3 homologs are present in Bikonta such as Stramenopiles (ciliates, brown algae, and *Plasmodium*) and Cryptophytes but could not be found in the *Ciona* or human genomes. DC3 grouped into a clade closer than LC4 to CNB/calaxin/NCS (Figure 5). Intriguingly, BLASTP searches using recent genomic information on the chlorarachniophyte *Bigelowiella natans* did not detect orthologs of *Chlamydomonas* LC4 or DC3. The protein with highest similarity was CaM (ID 54077), although ultrastructural observation of the flagella clearly shows the presence of outer arm dynein [123]. LC4 was also absent from *Plasmodium* (Apicomplexa).

Both CN-B and NCS have been found in animals and fungi [124] but do not appear in plants. In plants, the CNB-like protein (CBL) family represents a unique group of calcium sensors and plays a key role in intracellular Ca^2+^ signaling [124]. CNB-like proteins in plants are most closely related to CNB and NCS proteins in animals and fungi (Figure 5). Proteins in *Chlamydomonas* (ID391130) and in *Paramecium* (GSPATP9660001) are grouped with CNB-like protein. Separation of these proteins from the CNB group is supported by the bootstrap value (986/1,000).

**Figure 6:**
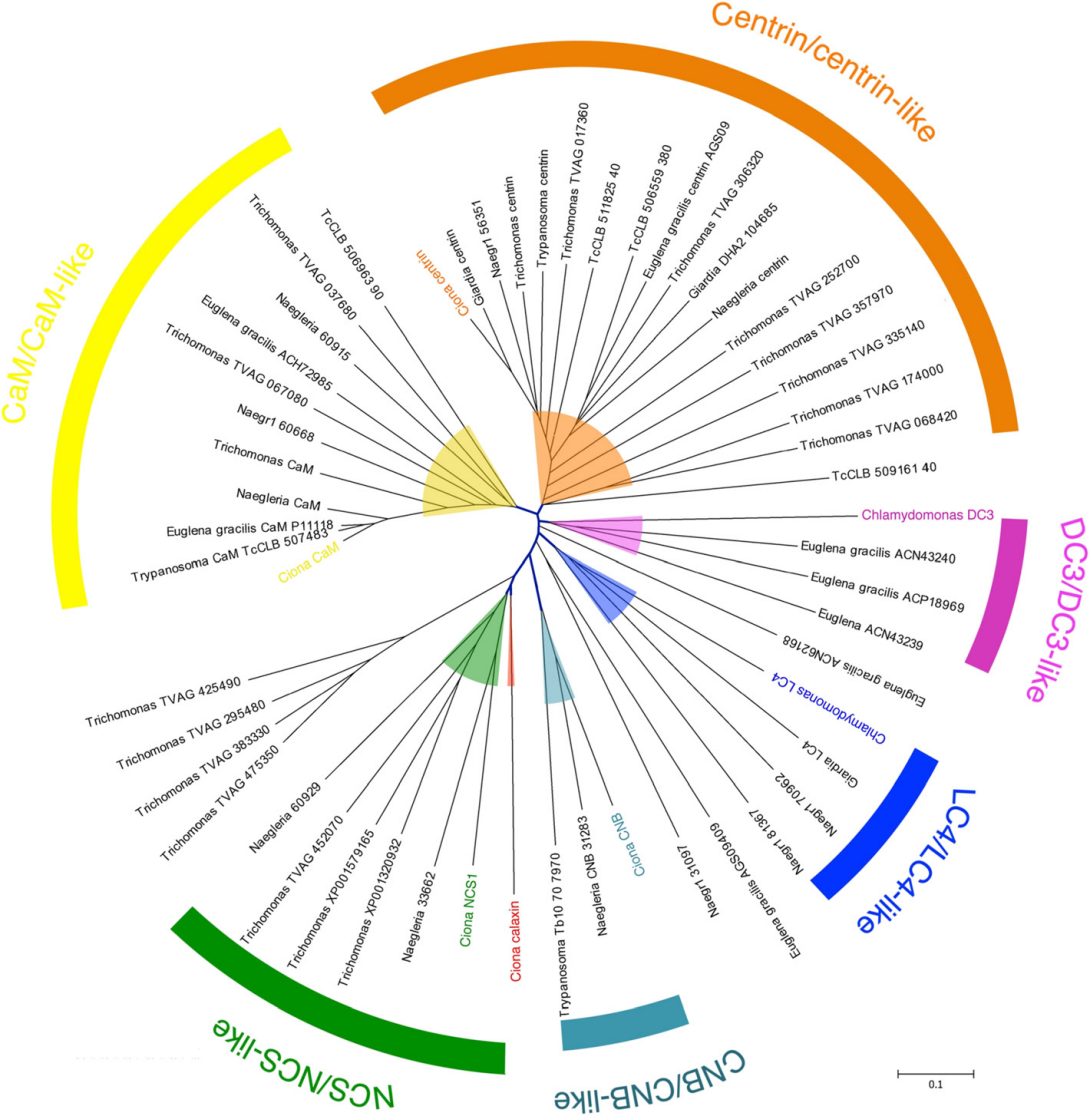
**Phylogenetic analysis of homologs of Ca** ^**2+**^ **sensor proteins in Excavata.** Proteins (EF-hand proteins, length less than 350 amino acids) were searched against genomes of each excavate by BLASTP and those with E-value <e^−9^ were aligned with *Ciona* or *Chlamydomonas* Ca^2+^-sensors by CLUSTALW. An unrooted tree was drawn by MEGA5. Branches of each Ca^2+^-sensor are highlighted by colors. The protein sequences (with accession numbers indicated) were obtained from DDBJ/EMBL/GenBank, or from the genome browsers shown in the legend of Figure 5.

The supergroup Excavata includes eight taxa [125-128]. Phylogenetic analysis supports the monophyly of the Excavata [128] which consists of two major groups, Discoba and Metamonada. An additional organism, Malawimonas, may also be included as a genus in the Excavata. Discoba includes four phyla, Jakobida, Euglenozoa (for example, *Euglena*, *Trypanosoma*), Heterolobosea (for example, *Naegleria*), and Tsukubamonadida. The Metamonada includes amitochondriate flagellate Fornicata (for example, *Giardia*), Parabasalids (for example, *Trichomonas*), and Preaxostyla [126]. Although Excavata are often considered the extant organisms closest to the ancient eukaryotes, there are debates concerning their phylogenetic position.

**Figure 7:**
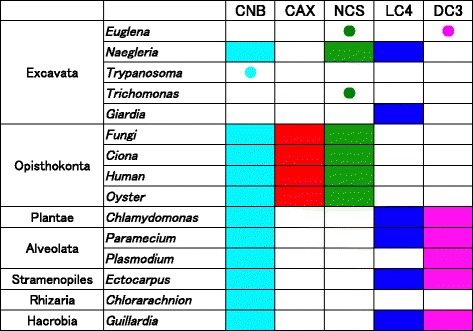
**Distribution of Ca** ^**2+**^ **sensor proteins in eukaryotes.** Based on the BLASTP search and the phylogenetic analyses in Figures 5 and 6, occurrence of each Ca^2+^ sensor in eukaryotic groups is summarized. Occurrence is indicated in the same colors as used in Figures 5 and 6. Closed circles in a specific color represent an occurrence of homologs with weak bootstrap support.

Analysis of Ca^2+^ sensors in Excavata leads to an interesting point of view concerning the evolution of Ca^2+^ sensor proteins (Figures 5, 6 and 7). First, both *Giardia lamblia* (XP_001705117) and *Naegleria gruberi* (ID 70962) contain clear orthologs of *Chlamydomonas* LC4 (Figure 5). Second, *Naegleria* has clear orthologs of NCS-1 and CNB (Figure 5). Third, several excavate species have multiple proteins with similarity to CNB, NCS-1, LC4, or DC3 (Figure 6), although they could only be grouped into each Ca^2+^ sensor family with weak bootstrap support. Euglena has three DC3-like proteins. *Naegleria* has a LC4-like protein. *Trypanosoma* Tb10707970 is a CNB-like protein. *Trichomonas* has three NCS-1-like proteins. There are other proteins in *Trichomonas*, *Naegleria*, and *Euglena* that are similar to, but could not be grouped with, any ciliary Ca^2+^ sensors (Figures 6 and 7). These features of Ca^2+^ sensors or their homologs in Excavata suggest that duplication and divergence of Ca^2+^ sensors occurred in this supergroup.

**Figure 8:**
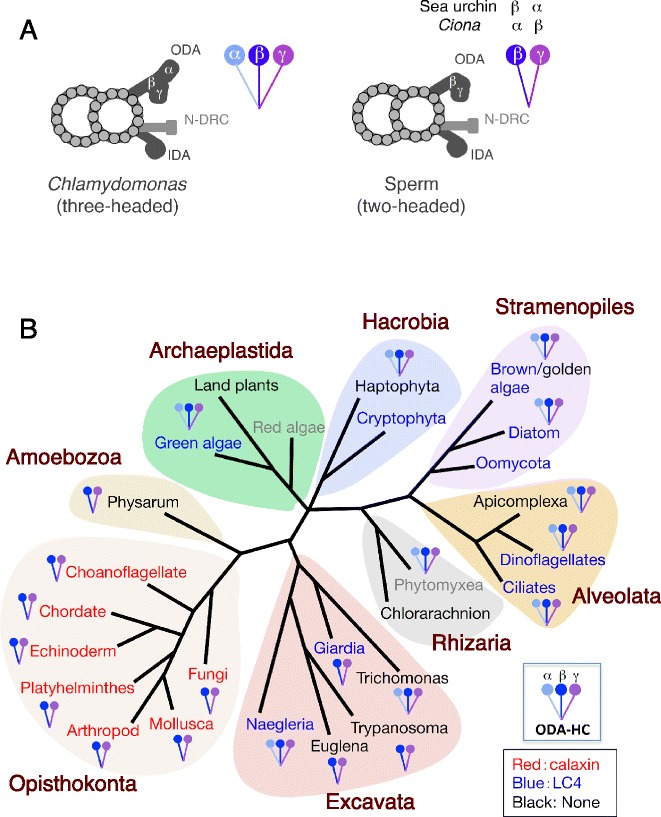
**Structure of outer arm dynein and its Ca** ^**2+**^ **sensor across eukaryotic groups. (A)** Schematic representation of the number of dynein heavy chains and the morphology of outer arm dyneins observed by electron microscopy. *Chlamydomonas* outer arm dynein is composed of three heavy chains, α, β, and γ. *Ciona* outer arm dynein has two heavy chains homologous to the *Chlamydomonas* β and γ chains. The α and β heavy chains in *Ciona* and the β and α heavy chains in sea urchin correspond to *Chlamydomonas* β and γ, respectively. ODA, outer arm dynein; IDA, inner arm dynein; N-DRC, nexin link/dynein regulatory complex. **(B)** Distribution of two-headed or three-headed outer arm dynein, and calaxin or LC4, across eukaryotic groups. The occurrence of calaxin or LC4 is indicated in red or blue, respectively, in the name of the group. A group name in black or gray indicates the lack of both calaxin and LC4, or not enough genomic information, respectively. The references for the EM images of the axonemes and the outer arm dynein are as follows: *Naegleria* [146]; *Euglena* [176,177]; *Trypanosoma* [66,67]; *Giardia* [144]; *Trichomonas* [147]: amoebozoan (*Physarum*) [101-103]; choanoflagellate (*Codosiga botrytis*) [178]; chordate (*Ciona intestinalis* and human) [62,88]; echinoderm (sea urchin: *Colobocentrotus atratus*) [1,3]; platyhelminthes (*Dugesia tigrina*) [68,179]; arthropod (*Exechia seriara*) [180]; Mollusca (*Crassostrea gigas*) [181]; chytrid fungus (*Rhizophlyctis*) [182]; green alga (*Chlamydomonas*) [137]; diatom (*Biddulphia levis*) [183]; golden alga (*Ochromonas*) [116]; ciliate (*Tetrahymena pyriformis*) [184]; dinoflagellate (*Wolszymkia micra*) [185]; apicomplexan (*Plasmodium*) [141]; chlorarachnion (*Bigelowiella natans*) [123]; haptophyte (*Chrysochromulina*) [186]; and phytomyxean (*Plasmodiophora brassicae*) [140].

### Ca^2+^ sensors appear to evolve with dynein heavy chains

As described above, *Ciona* and *Chlamydomonas* use distinct Ca^2+^ sensors for outer arm dynein. The molecular properties of these two proteins differ from each other, and this might be related to the difference in Ca^2+^-dependent regulation of flagellar motility. BLAST searches using genomic information from several organisms indicate that calaxin is an opisthokont-specific protein. Orthologs of *Chlamydomonas* LC4 are distributed in Archaeplastida, Alveolata, Stramenopiles, Cryptophytes, *Giardia* and *Naegleria*, but not in Opisthokonta or the excavates *Euglena* and *Trypanosoma*.

Ca^2+^ sensors directly act on the motor subunits of outer arm dynein. The heavy chains of outer arm dynein are phylogenetically classified into ODAα and ODAβ families [129]. The ODAα family includes the *Chlamydomonas* γ heavy chain, the *Ciona* β heavy chain, and the sea urchin α heavy chain, all of which are located at the innermost part of the outer arm [130,131]. The ODAβ family includes the *Chlamydomonas* α and β heavy chains, the *Ciona* α heavy chain, and the sea urchin β heavy chain^a^.

It is known that the number of heavy chains of outer arm dynein is two in metazoan sperm but three in *Chlamydomonas* and ciliates [132-136]; from the molecular structure of dynein, they are called two-headed and three-headed. EM images of cross sections of the axonemes enable analysis of the number of heavy chains of outer arm dynein (Figure 8A; [133]). The outer arm of a *Chlamydomonas* mutant lacking the α heavy chain lacks the outermost part and appears similar to the outer arm of sperm flagella [137,138], indicating that the outermost part corresponds to the α heavy chain. Other observations by transmission electron microscopy (TEM) [138] or cryo-electron tomography [130,131] indicate that the innermost part and the center part of the TEM image is composed of the γ and β heavy chain in *Chlamydomonas*, respectively. Following the idea of Mohri *et al*. [133], the number of heavy chains could be predicted from the morphology of outer arm dynein observed by TEM (Figure 8A). I surveyed published TEM images of outer arm dyneins in several organisms. It is intriguing to note that the number of dynein heads and the Ca^2+^ sensor used for regulation of outer arm dynein turn out to be well correlated (Figure 8B).

**Figure 9:**
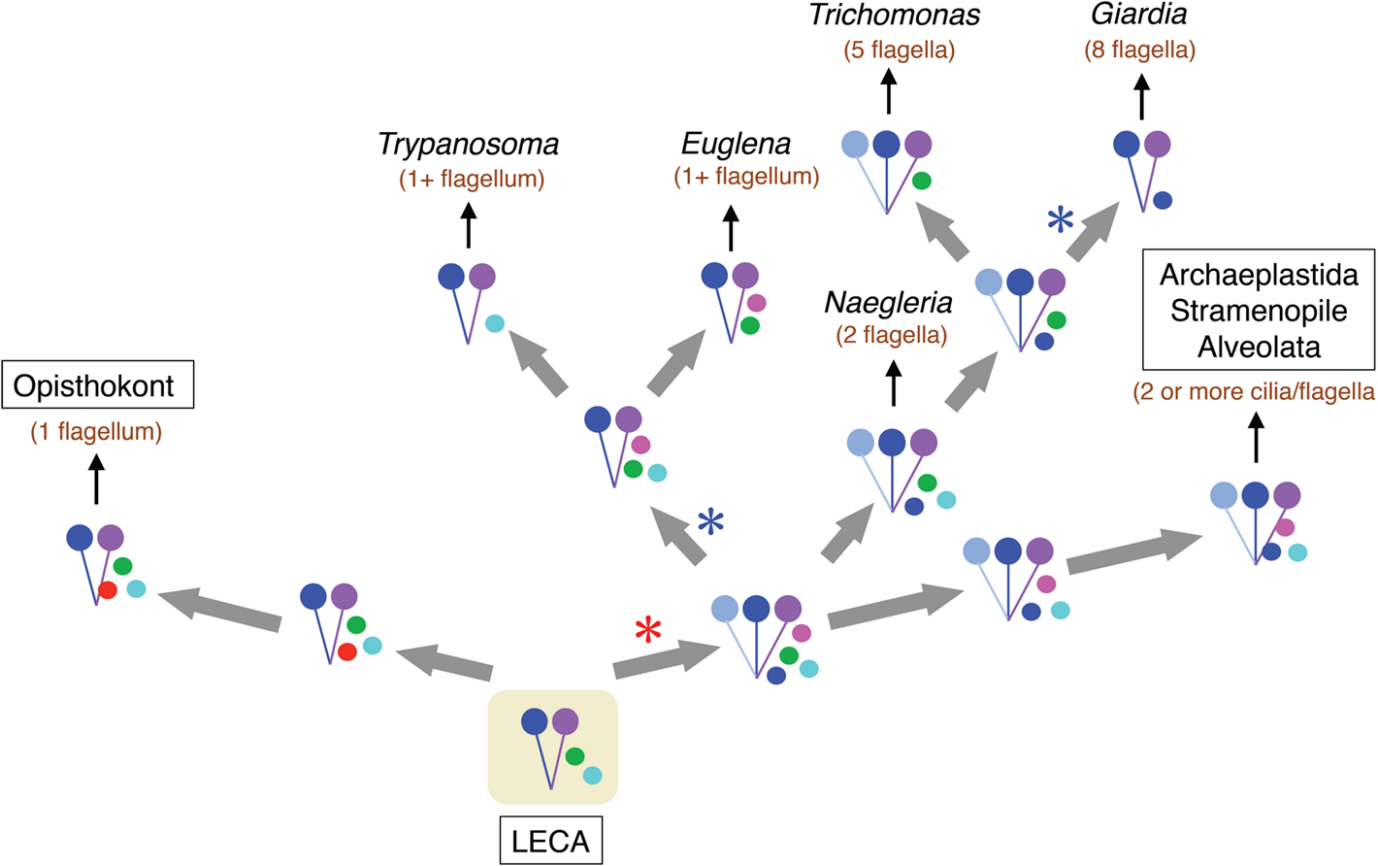
**A possible model for the evolution of, and diversification in, the structures of outer arm dynein and corresponding Ca** ^**2+**^ **sensors during eukaryotic evolution.** The model is based on analyses of the structures of outer arm dynein (two-headed, three-headed) and the types of Ca^2+^-sensor in each group of eukaryotes. It is assumed that the heavy chains and Ca^2+^-sensors of outer arm dynein of the last eukaryotic common ancestor (LECA) preceded duplication, and that duplication and divergence of Ca^2+^-sensors occurred at an early stage of eukaryotic diversification. The model is arranged so that the positions of eukaryotic groups match with widely accepted phylogenetic relationships [128,158]. The number of cilia/flagella per cell is also indicated in parenthesis (brown letters). Note that the numbers of cilia/flagella in *Euglena* and *Trypanosoma* are indicated as ‘1+,’ since these organisms are considered to have been biflagellates but lost or largely degenerated one of the two flagella during evolution. In this model, duplication of dynein heavy chain occurred at the root of the bikont lineage. Duplication and divergence of Ca^2+^-sensors would have already occurred in the ancestral organisms that contained three-headed dynein. An ancestral organism containing three-headed dynein might have recruited LC4-like sensors or CNB/NCS-like sensors and then branched into the Metamonadan (*Trichomonas* + *Giardia*) and Discoban lineages. Loss of dynein heavy chains would have occurred in *Giardia* and the Euglenozoa. Red or blue asterisks represent duplication or loss of a dynein heavy chain, respectively. Colored dots next to the two- or three-headed dyneins represent Ca^2+^-sensors (red, calaxin; blue, LC4; magenta, DC3; green, NCS; cyan, CNB). In the lineage of opisthokonts or Archaeplastida/Stramenopile/Alveolata, calaxin, LC4 or DC3 is demonstrated to be bound to the dynein heavy chain, although it is not known whether Ca^2+^-sensors in Excavates or any of the hypothetical ancestors could bind to the dynein or not.

It is believed that the two heavy chains of the ODAβ family resulted from gene duplication [139], but the exact phylogenetic position of the duplication is not clear. The biflagellated swarm cells in the amoebozoan *Physarum* possess 9+2-structured flagella. Cross sections of *Physarum* axonemes suggest that the outer arm dynein is two-headed [101-103], like those in opisthokonts. However, the presence of calaxin and the number of heavy chains in the outer arm dynein remain unclear because of the lack of a genome sequence. Recent genome information reveals no gene similar to *Chlamydomonas* LC4 or DC3 in the chlorarachnion *Bigelowiella natans*. The number of heavy chains is possibly three judged from an EM image [123]. Another cercozoan, *Plasmodiophora brassicae*, apparently possesses three-headed outer arm dynein [140], but no genomic information is available. Ciliates, such as *Paramecium* and *Tetrahymena*, have three-headed outer arm dynein and a gene orthologous to *Chlamydomonas* LC4. However, another group of Alveolata, the Apicomplexa, shows a different feature; the axonemes of *Plasmodium berghei* have normal 9+2 structure with three-headed outer arm dynein [141]. It is not clear whether *P. berghei* has LC4 since the genome sequence of this organism is not available. The gregarin *Lecudina tuzetae* has a 6+0 structured axoneme, but the detailed structure of the outer arm dynein is unclear from the available EM images [142].

Six species in the Excavata were available for prediction of the number of heavy chains from EM images. First, the euglenozoan species *Euglena*, *Leishmania*, and *Trypanosoma* show a two-headed shape of outer arm dynein. The genome sequences reveal that neither *Euglena* nor *Trypanosoma* have LC4. Second, *Giardia* has an LC4 homolog in the genome. EM images, however, are very close to those of two-headed outer arm dynein [143,144]. This might be because *Giardia lamblia* is a fast-evolving parasitic species, leading to an error in phylogenetic analysis due to long-branch attraction (LBA) [145]. Lastly, the outer arm dyneins of two excavate species, *Naegleria gruberi* and *Trichomonas vaginalis*, appear three-headed, although little TEM data with clear images of outer arm dynein is available [146,147].

### Eukaryote evolution in view of outer arm dynein and its calcium sensors

The structure of the axoneme and the regulation of ciliary and flagellar motility are basic aspects of all major eukaryotic groups and undoubtedly one of the ancestral features of eukaryotes [148-151]. There are three hypotheses for how cilia were acquired in the last eukaryotic common ancestor (LECA): endosymbiosis of a Spirochete and an Archaebacterium [152], viral infection [153], and autogenous origin [153] (see reviews [149,154]). The latter hypothesis is widely accepted at present. During overall evolution of cell motility, ciliary movement and amoeboid movement were selectively or cooperatively used depending on the body plan of the organisms. In the most probable LECA unicellular organism, both ciliary and amoeboid locomotion systems appear to have been used [151]. Ancient flagella are considered to be used for attachment to a substrate and to pull the organism by gliding. It is possible that flagella then acquired regulatory systems for directed, tactical, or avoiding movement with high speed, with the aid of extracellular signaling molecules such Ca^2+^; examples of such regulated movement are reversal of bend propagation and altering of flagellar waveforms (Figure 1). In this case, as much evidence indicates, Ca^2+^-dependent regulation of the outer arm dynein is thought to be critical. During diversification, some organisms lost components of the axoneme. For example, loss of outer arm dynein is probably due to the loss of a requirement for rapid and/or extensive reorientation of the cell. Other organisms have lost motile flagella or cilia, probably due to disuse of their motility, in, for example, reproduction. The former include the gregarin *Lecudina tuzetae*, *Breviata*, fern, moss, eel, and insects like *Acerentomon microrhinus*, and the latter include nematodes, crustaceans, and angiosperms [154,155].

Taking into account the fact that cilia have been inherited through the major pathways of eukaryotic evolution, I here propose a hypothesis for eukaryotic evolution based on phylogenetic analyses of Ca^2+^ sensors and the number of dynein heads. The most evident feature is that the majority of opisthokonts show two-headed outer arm dynein with the Ca^2+^ sensor calaxin, whereas the majority of bikonts (Archaeplastida, Stramenopiles, Alveolata, and some (but not all) Excavata) have three-headed outer arm dynein with *Chlamydomonas* LC4-type Ca^2+^ sensors. Excavata robustly emerge between unikonts and Archaeplastida/Hacrobia/Stramenopiles/Alveolata/Rhizaria and form a monophyletic supergroup [128]. Several phylogenetic analyses of diverse eukaryotes have led to the idea that the eukaryotic root could be set at the base between unikonts and bikonts [156-158], but this is still controversial [158-162].

The Excavata is certainly a supergroup that could provide key clues to understanding the evolution of dynein and its Ca^2+^ sensors and shed light on the origin of Ca^2+^-dependent regulation of cilia and flagella. A phylogenetic analysis in this study showed that excavates had already evolved several Ca^2+^ sensors, including those with similarities to extant Ca^2+^ sensors. Based on the widely accepted relationship among excavate species [128,158], a possible pathway could be considered with respect to evolution of dynein structure and Ca^2+^ sensors (Figure 9). This model is based on the hypothesis that the LECA had two-headed dynein and that Ca^2+^ sensors were duplicated in the initial stage of eukaryotic evolution and became divergent (and then possibly became functional) during evolution. Loss of dynein heavy chains or Ca^2+^ sensors in Excavata, possibly by reduction of genomes in obligate parasites [143,163], is also taken into consideration.

The duplication of dynein heavy chains would have occurred at the root of the bikont lineage (Figure 9). From the strong bootstrap supports (Figure 5), it appears that three-headed dynein might have recruited LC4 in the last common ancestor of bikonts, which would be involved in the diversification in Metamonada (*Trichomonas* and *Giardia*). Similarly, CNB/NCS-like Ca^2+^ sensor homologs must have existed in the last common eukaryotic ancestor. Another route for Discoba diversification might have involved retentions of CNB/NCS-like Ca^2+^ sensors.

Excavates show a variety in the number of motile flagella per cell. For example, the euglenoids *Trypanosoma brucei* and *Euglena gracilis* are biflagellate but one of the two flagella is highly reduced. There are two flagella in *Naegleria gruberi*, five flagella in *Trichomonas vaginalis*, and eight flagella in *Giardia lamblia* (see Figure 9). It is worth pointing out that the excavate species bearing a single motile flagellum, that is, *Euglena* and *Trypanosoma*, have two-headed dyneins; *Giardia* is the only excavate with two-headed dynein and multiple flagella (Figure 9). The only other eukaryotic group containing organisms (or cells) with a single motile flagellum is the Opisthokonta.

The Amoebozoa, *Physarum polycephalum* and *Breviata anathema*, originally grouped into unikonts [125], bear two basal bodies. It has therefore been debated whether Amoebozoa and Opisthokonta can be monophyletically grouped [157,164]. *Physarum* has one long and one short flagellum connected to two basal bodies, and *Breviata anathema*, a small amoeba-like cell, has a single flagellum from each of the two basal bodies. The presence of two basal bodies is proposed as one of the characteristics of bikonts [165]. From TEM images of axonemes, *Physarum* appears to have two-headed outer arm dyneins (Figure 8), which is a common aspect of opisthokonts [133]. *Breviata* does not have outer arm dynein [100], meaning there is no evidence for its grouping based on the criterion of the structure of outer arm dynein. It would be intriguing to search for calaxin (also TNDK-IC and CMUB, see above and [166]) in organisms that have been under debate in terms of classification into bikonts or unikonts.

New genes with novel functions are evolved by gene duplication [167]. Several models have been proposed for mechanisms of how new protein functions evolve through gene duplication and divergence [168]. Recruitment of functional Ca^2+^ sensors seems particularly important in cilia and flagella because they participate in gamete motility, essential for the success of reproduction in most organisms. For Ca^2+^ sensors of outer arm dynein, the functions of calaxin and *Chlamydomonas* LC4 regulate the motor activity in flagella, but their response to Ca^2+^ concentration is different. The distribution of these Ca^2+^ sensors in extant species in eukaryotes is described in the present paper. Calaxin and LC4 appear to be preserved in Opisthokonta and the majority of bikonts (Archaeplastida, Stramenopiles, and Alveolata), respectively.

It is possible that these proteins became preserved after protein evolution by gene duplication and divergence because of their specific functions in the interaction with the cytoskeleton and the regulation of a molecular motor. The module-dominant conservation, as seen in axonemes [166], is possibly because of the need for preservation of multiple proteins in this cytoskeletal architecture. No biochemical evidence has been obtained for the localization or functions of Ca^2+^ sensors, except *Ciona* calaxin and *Chlamydomonas* LC4. To learn whether evolution of proteins by gene duplication and divergence accompanies or precedes innovation of protein function, it would be fascinating to examine the interaction of an ancient calaxin with microtubules or dyneins.

## Conclusions

Conversion from asymmetric to symmetric movement at high concentrations of Ca^2+^ requires outer arm dynein in *Chlamydomonas* flagella. Conversion to an asymmetric waveform in sperm flagella is also performed by outer arm dynein at high Ca^2+^ concentration. Thus, the functions of outer arm dynein are regulated by Ca^2+^ sensors at high concentrations of Ca^2+^ in both *Chlamydomonas* and sperm flagella. Recruitment of Ca^2+^ sensors to outer arm dynein might have made it possible for the organisms to respond to ‘high’ Ca^2+^ to modulate flagellar waveforms to change their direction of movement, although the directions of conversion of waveforms are a mirror-image of each other in *Chlamydomonas* and sperm.

In this paper, it is suggested that the duplication and divergence of Ca^2+^-sensors might have occurred at an early stage in eukaryotic evolution. The clear distinction in dynein structure and Ca^2+^ sensors between opisthokonts and bikonts, and their heterogeneity in Excavata, suggests an important role of ciliary regulation in eukaryotic evolution. It is unclear, however, if Ca^2+^ sensors in Excavata really function in the regulation of outer arm dynein. Outer arm dynein in *Trypanosoma* is essential for tip-to-base movement, which is induced by ‘low’ intracellular Ca^2+^. Loss of outer arm dynein results in a defect of tip-to-base movement in response to low intracellular Ca^2+^. This feature of Ca^2+^ regulation of outer arm dynein is distinct from that observed in *Chlamydomonas* and *Ciona. Trypanosoma* and *Naegleria* have CaM in flagella called flagellar CaM or CaM-1. CaM is localized in paraflagellar rods and regulates their assembly in *Trypanosoma* [169]. However, it is unclear whether CaM is localized to the outer arm dynein or other axonemal structures. Further studies are necessary to elucidate the role of Ca^2+^-binding proteins in the regulation of the outer arm dynein in Excavata.

Calaxin was acquired in Opisthokonta and may participate not only in the regulation of fluid flow mediated by cilia and flagella but also in other phenomena that characterize opisthokonts, such as cell polarity, differentiation of nerve cells, and establishment of body plan. The first definition of Opisthokonta by Cavalier-Smith [170], that is, organisms having posterior flagella to propel cells forward, may be related to the position of the sperm acrosome in the anterior part where sperm adhere and fuse with the counterpart gamete egg. The corresponding portion of *Chlamydomonas*, the mating structure, sits between two flagella. Both *Chlamydomonas* and sperm move forward with these mating structures at the leading edge (Figure 1). Differentiation of the sperm acrosome accompanies the localization of the Golgi apparatus and vesicles at the anterior of the head [171,172]. The Golgi apparatus and vacuoles are likely to locate near the flagella of *Chlamydomonas* [173], implying that the intracellular compartments for gamete recognition are reversely positioned relative to the positions of basal bodies between sperm and *Chlamydomonas*. Although it is not known whether this difference in cell polarity is connected to the mirror-image of Ca^2+^-dependent regulation between these cells, experiments such as knockout of the calaxin gene in metazoa might give important insights into the evolutionary relationship between cilia and organisms.

The present study implies early events in the diversification of Ca^2+^ sensors for outer arm dynein during evolution, but connections of the phylogenetic view of outer arm dyneins to the function or motility of cilia and flagella have not been completely clarified. Most of the discussion in this paper is based on the assumption that orthologous proteins conserve their function across species, but this is not always the case. For solving such problems, it is evidently necessary to confirm whether the proteins used in phylogenetic analyses in the present study are localized and bound to the ciliary or flagellar axonemes and function as Ca^2+^ sensors of outer arm dynein. The precise number of heads must also be determined by observation with cryo-electron tomography.

## Endnote

^a^The nomenclatures of dynein heavy chains are complicated because they were originally named according to the electrophoretic mobility on an SDS-gel (see Table 2; refs [56,57,166,174]).

## Abbreviations

EM: electron microscopy
IC: intermediate chain
LC: light chain
NCS: neuronal calcium sensor
RNAi: RNA interference
